# Transformation of T-Cell Acute Lymphoblastic Lymphoma to Peripheral T-Cell Lymphoma: A Report of Two Cases

**DOI:** 10.1155/2018/9191582

**Published:** 2018-02-28

**Authors:** Michael Markow, Abu-Sayeef Mirza, Lia Perez, Haipeng Shao, Pedro Horna, Claudio Anasetti, Lubomir Sokol, Mohammad O. Hussaini

**Affiliations:** ^1^Department of Pathology, Ohio State University, Columbus, OH, USA; ^2^Department of Internal Medicine, University of South Florida, Tampa, FL, USA; ^3^Bone Marrow Transplant Program, Moffitt Cancer Center, Tampa, FL, USA; ^4^Moffitt Cancer Center, Tampa, FL, USA; ^5^Mayo Clinic, Rochester, MN, USA

## Abstract

Nonhepatosplenic/noncutaneous *γδ* peripheral T-cell lymphoma (NHNC*γδ* PTCL) represents a miscellaneous group of unrelated T-cell lymphomas of which only isolated cases have been reported. We describe two cases of transformation from T-lymphoblastic leukemia/lymphoma to NHNC*γδ* PTCL. Transformation into more aggressive disease is a rare event in T-cell lineage-derived hematologic malignancies compared to B-cell neoplasms. Nevertheless, both of our cases involved relapse as PTCL manifested with skin involvement and an overt shift from blastic morphology to large granular leukemia-like mature T cells. Among other notable molecular characteristics, expression of immature markers such as TdT was lost in both cases. Based on cytogenetics, phenotype, and morphology, both patients represent a novel phenomenon of clonal transformation from T-ALL to PTCL which has rarely been reported in the literature. Such transformation may carry important diagnostic and biological implications.

## 1. Introduction

Peripheral T-cell lymphomas (PTCLs) comprise a heterogeneous group of indolent and aggressive T-cell lymphomas that confer a variable prognosis [1]. T-cell receptor gamma-delta (TCR*γδ*) PTCLs are characterized based on their expression of *γδ* glycoproteins within the TCR complex rather than more common alpha-beta (*αβ*) glycoproteins identified in normal T-cells and in most T-cell neoplasms [2]. *γδ* T-cells are subdivided into V*δ*1 and V*δ*2 subtypes and have demonstrated capacity for cytotoxicity, memory, and antigen presentation [3]. Mature *γδ* PTCLs have no clear etiology to date and can be divided into three categories based on location: hepatosplenic (HSTL*γδ*), cutaneous (PCTCL*γδ*), and nonhepatosplenic/noncutaneous (NHNC*γδ* PTCL). HSTL*γδ* and PCTCL*γδ* both carry a similarly dismal prognosis [4].

NHNC*γδ* PTCL represents a miscellaneous group of unrelated T-cell lymphomas of which only isolated cases have been reported; thus, characterization remains limited. Reported sites of involvement include lymph nodes and mucosal sites (nasopharynx and intestine), as well as the larynx, thyroid, lung, breast, and testis [5–8]. NHNC*γδ* subtypes of PTCLs generally behave more aggressively compared to *αβ* counterparts with an exception of *γδ* variant of T-cell large granular lymphocyte (LGL) leukemia [9].

No cytogenetics findings unique to NHNC*γδ* PTCL have been reported [10]. i7q, if detected, is not specific to *γδ* PCTLs [11]. Gene sequencing of *γδ* PCTLs has demonstrated conservation of *LGR4*, *C3AR1*, and *SCARF2* among all *γδ* PTCLs [12]. Notably, the NHNC*γδ* PTCLs had *CCL19*, *MMP9*, and *UBD* mutations, whereas HSTL*γδ* PCTLs did not. These mutations were also strongly conserved in the *αβ* PTCLs studied. Although there is evidence that some variants of *γδ* PTCLs respond *in vitro* to aurora kinase inhibitors, among all PTCLs, those with TCR*γδ* have the worst prognoses [13–15].

As opposed to PTCL, the expression of *γδ* TCR is relatively common in (up to 50%) in T-lymphoblastic leukemia/lymphoma (T-ALL). Clinical features are similar to those that express *αβ* TCR, and outcomes are comparable [8]. Here, we describe a case of transformation from T-ALL to NHNC*γδ* PTCL. Despite being rarely reported, we also report a second case of PTCL arising in a patient with prior T-ALL. Such transformation may carry important diagnostic and biological implications.

## 2. Case Presentations

### 2.1. Patient 1

**Figure 1:**
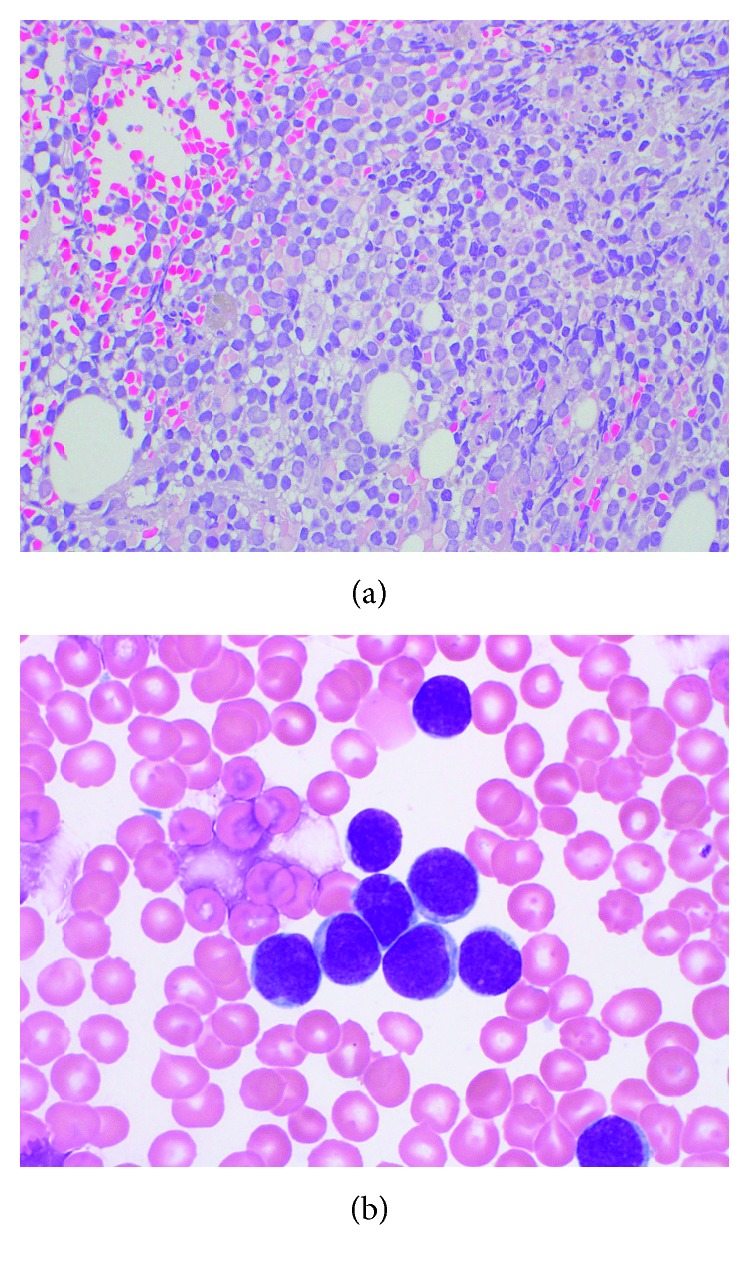
Patient 1, bone marrow with T-cell ALL: (a) H&E core biopsy, 500x; (b) aspirate cytology, 1000x.

**Figure 2:**
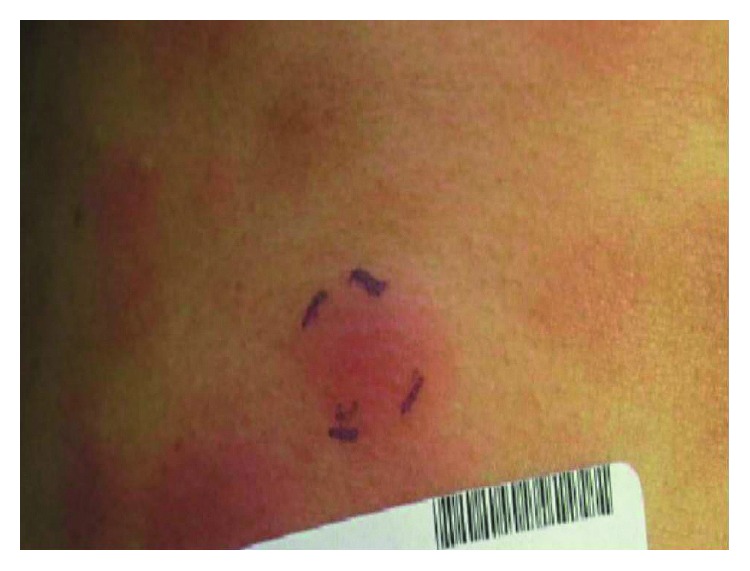
Patient 1, erythematous patches and plaques of PTCL distributed predominantly on torso and upper extremities.

**Figure 3:**
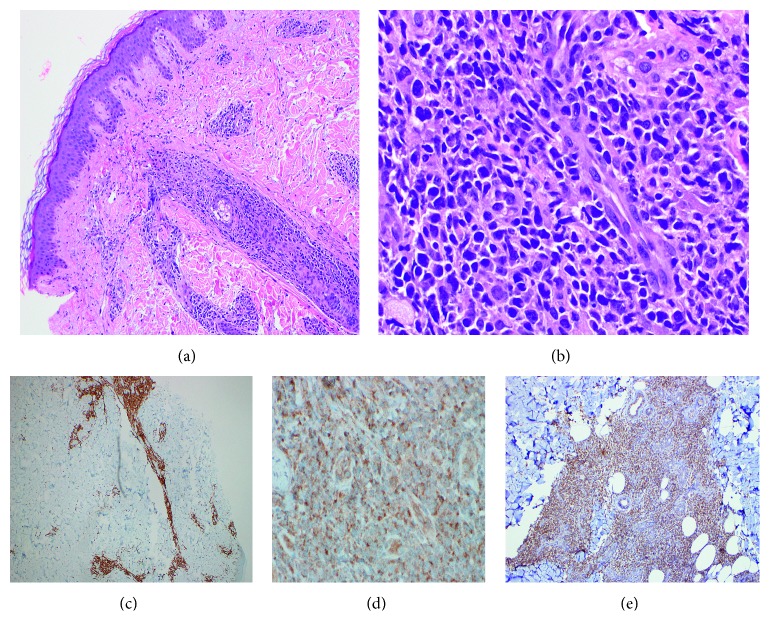
Patient 1, right flank skin with PTCL: (a) H&E, 100x; (b) H&E, 400x; (c) CD3, 100x; (d) TdT immunoperoxidase, 400x; (e) TCR gamma, 200x.

**Figure 4:**
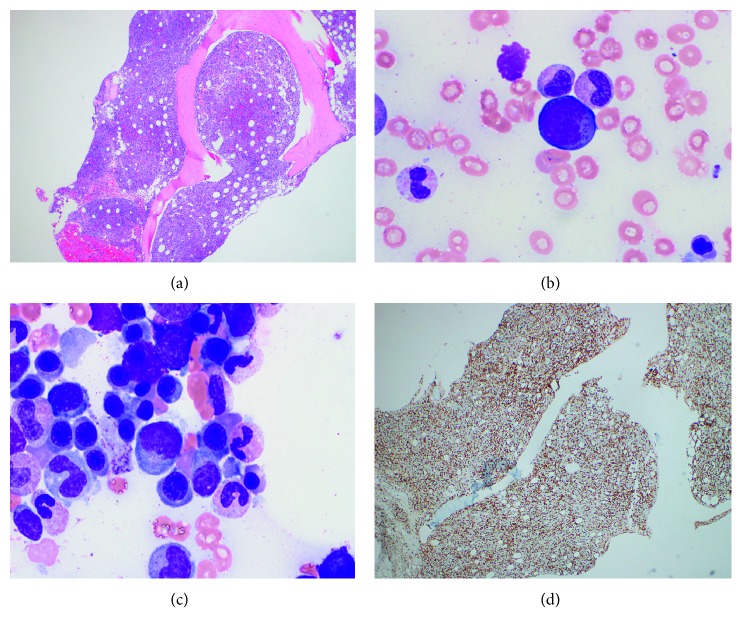
Patient 1, bone marrow with PTCL: (a) H&E, 40x; (b, c) aspirate cytology, 1000x; (d) CD3, 100x.

Patient 1 is a 31-year-old gentleman diagnosed with T-cell ALL after presenting with diffuse petechial rash, cervical lymphadenopathy, abdominal pain, and a WBC of 121 K/*μ*L with 40% blasts (Figure 1). Cytogenetics showed 46,XY,add(13) (p11.2)[3]/46,XY[17]. No BCR/ABL was detected by FISH; CSF was uninvolved. T-ALL was CD4+/CD8+, CD2+, CD3+, CD5+ (dim), CD7+, and TdT (dim). He was treated with vincristine, daunorubicin, pegylated L-asparaginase, and prednisolone achieving complete remission (CR). He was referred for hematopoietic stem cell transplant three months later which was delayed due to chemotherapy complications. During this period, he received leucovorin and glucarpidase. Prior to the transplant, a bone marrow biopsy was negative, but revealed clonal TCR *β* and *γ* gene rearrangements. In addition, CSF showed numerous LGL-like cells. The patient developed high grade fever and over one hundred erythematous plaques on his trunk, upper extremities, and lower extremities (Figure 2). Skin biopsy showed NHNC*γδ* PTCL (Figure 3) as did staging bone marrow biopsy (Figure 4). The malignant cells demonstrated mature chromatin, LGL-like morphology with prominent azurophilic granules resembling those seen in patient 2's PTCL (see below). They coexpressed CD4, CD8, CD56, cytotoxic markers, and *γ*- TCR. TdT was negative. Molecular studies of the PTCL demonstrated that both the T-ALL and PTCL shared identical clonal TCR rearrangement peaks for both TCR *β* and TCR *γ* (Table 1).

Six months after initial CR, the patient was initiated on ICE therapy and intra-Ommaya reservoir methotrexate (later switched to intrathecal cytarabine). The skin lesions and CSF involvement initially resolved; however, he developed fevers, new generalized skin *δγ*-PTCL, nodal/hepatic progression by radiology, and CSF recurrence consistent with progressive disease. Bone marrow transplant was deferred, and the patient was admitted for hyper-CVAD chemotherapy. However, the patient requested transfer despite the worsening condition. Current clinical status is unknown.

### 2.2. Patient 2

Patient 2, deceased, was a 63-year-old Caucasian female with a past medical history significant for stage III invasive ductal carcinoma, ER/PR/HER2 negative, with 1 of 9 axillary lymph nodes positive for carcinoma, diagnosed at age 52. She underwent a left segmental mastectomy (lumpectomy) procedure with axillary lymph node dissection, radiation treatment, and 4 cycles of doxorubicin and cyclophosphamide chemotherapy. Other medical problems included hypertension, hyperlipidemia, herpes zoster, childhood rheumatic fever, menopause, and hypersensitivity pneumonitis.

**Figure 5:**
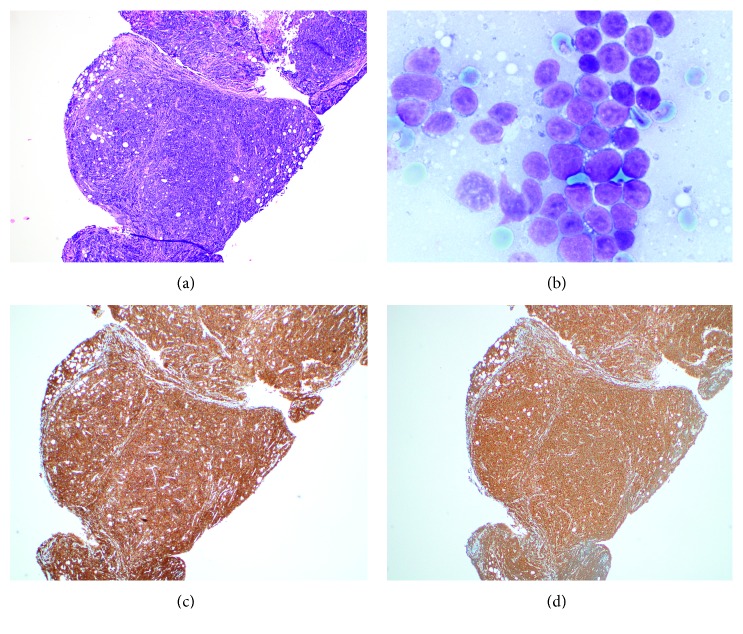
Patient 2; mediastinal mass with T-cell ALL: (a) H&E, 100x; (b) cytology, 1000x; (c) CD3, 100x; (d) TdT, 100x.

She initially presented with a mediastinal mass, dyspnea, and pericardial and recurrent right-sided pleural effusions. Biopsy of the mediastinal mass revealed blasts with cyCD3+, TdT+, CD4+/8+, CD7+ (Figure 5). TCR gamma gene rearrangement studies demonstrated clonal gene rearrangements. The peripheral blood and bone marrow were also involved.

**Figure 6:**
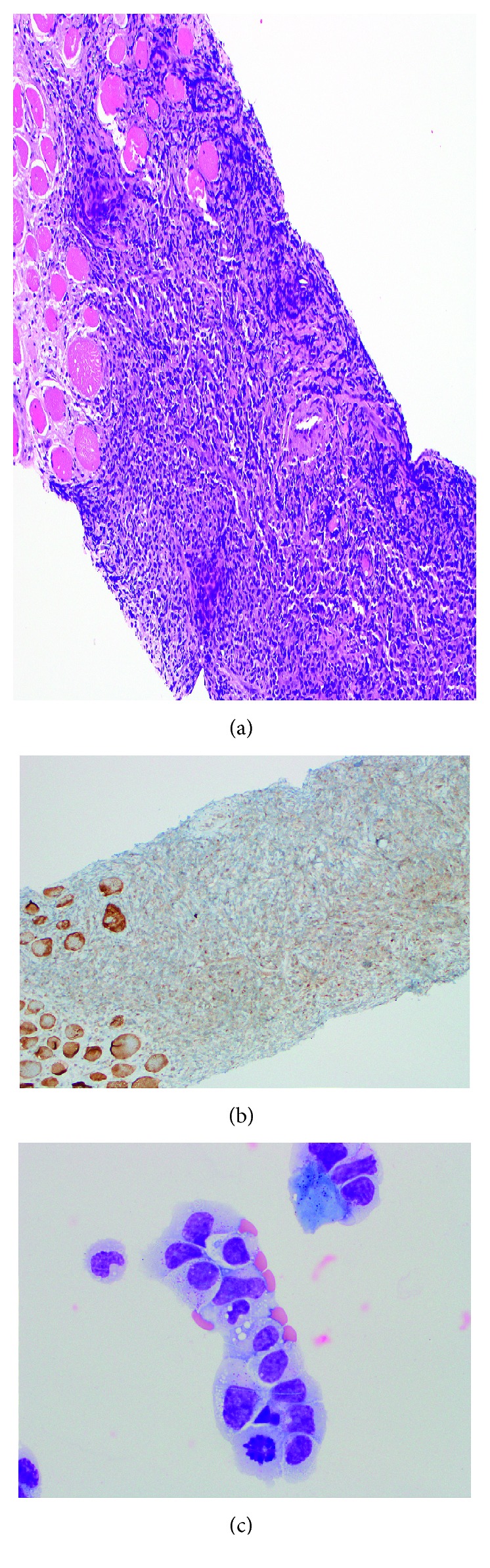
Patient 2: (a) flank mass with PTCL, H&E, 100x; (b) flank mass, TdT immunoperoxidase, 200x; (c) pleural fluid cytospin cytology, 500x.

She was treated with 18 cycles of hyper-CVAD followed by POMP maintenance for 18 months and remained in remission for nearly 4 years. Thereafter, she developed dyspnea, and radiographic imaging showed several simultaneous masses, including a 4.7 × 4.0 cm right atrial mass and a left flank mass, in addition to pleural effusions. Biopsy of the atrial mass was nondiagnostic showing myocardial and fibrous tissue. The chest wall (flank) mass was biopsied showing involvement by PTCL (Figure 6). Pleural fluid analysis showed involvement by CD3+ large lymphoid cells with markedly irregular nuclear contours, cytoplasmic azurophilic granules, lacking TdT and CD7, and showing positivity for CD8. Clonal TCR *β* and *γ* gene rearrangements were detected. Staging bone marrow biopsy showed no evidence of T-ALL.

However, clonal TCR *β* and *γ* gene rearrangements were detected with a possible similar peak to that detected in the pleural effusion sample (Table 2). Shared identical clonal TCR peaks were not noted between T-ALL and PTCL specimens, but comparison is limited by the fact that TCR testing was performed in different labs and may have used disparate primer sets and analysis parameters. The patient later received 2 cycles of ESHAP resulting in partial response. However, PET scan showed SUV of 11 in the cardiac area. Romidepsin salvage chemotherapy was administered. The patient continued to have large pleural effusions with respiratory issues and eventually opted for hospice. She died 4 months after her presentation with PTCL and 50 months after her original diagnosis of T-cell ALL, at age 64.

## 3. Discussion

In both cases, there was relapse as PTCL manifested with skin involvement and an overt shift from blastic morphology to “LGL-like” mature T cells with ample cytoplasm and abundant azurophilic granules. Phenotypically, expression of immature markers such as TdT was lost in both cases. Both patients reasonably represent a novel phenomenon of clonal transformation from T-ALL to PTCL which has rarely been reported in the literature. In patient 1, there is clear evidence of a clonal relationship as demonstrated by identical monoclonal TCR peaks. Also, both PTCL and T-ALL cases demonstrated the relatively immature CD4+/CD8+ immunophenotype. HSTL*γδ* are often CD4-/CD8-, recapitulating normal *γδ* T cells, and less commonly C8+/CD4-is seen. In other *γδ* PTCLs, CD4+/CD8− phenotype may be observed perhaps deriving from a subset of normal *γδ* T cells that harbor this phenotype [8]. However, dual CD4+/CD8+ has not been typically reported and is difficult to explain [16]. A clonal relationship is less clear for patient 2. However, the genesis of an entirely new PTCL in a patient with established T-ALL would be unusual indeed. TCR gene rearrangements cannot be reliably compared between the original T-ALL and PTCL due to their performance in differing laboratories.

Morphologically, *γδ* PTCLs are a heterogeneous group. However, in both our cases we find a “LGL-like” morphology with large cells with ample cytoplasm containing cytoplasmic granules further supporting the notion that a common phenomenon may be at play in these two cases. Furthermore, SNP microarray performed on patient 2's PTCL showed clonal alterations in 95% of the cells, but clonal evolution was seen in 41–55% of the cells suggesting further that this represents a type of transformation. Furthermore, homozygous interstitial deletion 14q32.2 was detected which houses *BCL11B*. *BCL11B* encodes a transcription factor necessary for normal T-cell development and has been implicated in T-ALL pathogenesis [17]. Another line of evidence that supports the idea that these cases may represent a novel phenomenon is the detection of aberrations occurring at similar genetic loci in both cases. In patient 1, karyotype showed 46,XY,add (13) (p11.2) [3]/46,XY[17] in the original T-ALL. In patient 2, SNP array performed on PTCL containing pleural fluid showed aberration at a similar locus: 13p11.1q24.21(34,474,059–115,670,586)x1-2.

If this truly represents transformation, we must consider the pathophysiology of such conversion. It may be postulated that chemotherapy may have forced the original T-ALL cells to undergo some degree of maturation. However, the disparate chemotherapy regimens in both cases, lack of similar conversions in other patients treated with these typical chemotherapy regimens, and temporal distance of relapse in patient 2 (3-4 years) do not support this speculation. More likely, acquisition of additional mutations in T-cell differentiation genes may have created a genetic context to allow for such transdifferentiation. This is supported by SNP array analysis performed on patient 2's PTCL showing clonal evolution. Another possibility is that malignant clones of T-ALL and PTCL developed separately from a common precursor sharing an initial transforming event. Subsequently, T-cell ALL and PTCL could evolve secondary to additional and separate transforming events.

The transformation into more aggressive disease is a rare event in T-cell lineage-derived hematologic malignancies compared to B-cell neoplasms. A patient with *γδ*-variant of LGL leukemia who developed aggressive ALL-like disorder after about 20 years of an indolent course has previously been reported [16]. Interestingly, cytology of leukemic cells during aggressive phase of disease did not differ significantly from LGLs assessed during indolent phase of disease. However, LGLs of the aggressive leukemia revealed very high Ki-67 proliferation index of 80%, and SNP array showed multiple genetic abnormalities most probably implicated in the transformation. This report supports our hypothesis that transformation in T-cell lymphomas/leukemia is a very rare event and that multiple acquired somatic mutations secondary to therapeutic or environmental factors likely facilitated the transformed phenotype.

Besides the pathophysiology, the clinical and biological nature of these transformed PTCLs can be considered challenging. Should these cases be considered “ALL-like” disorders given their putative clonal derivation from T-ALL or do they represent a new entity with unique biology? The second case was considered to be a new entity with unique biology, hence the aggressive, chemotherapy refractory course that resulted.

In summary, we report two cases of NHNC PTCL arising in patients with established diagnoses T-ALL which, in at least one case, is clonally related. This raises the possibility of a novel pathologic phenomenon with associated diagnostic and biological implications.

## Figures and Tables

**Table 1:** Patient 1 pathological findings, by site.

Site (days from original diagnosis)	Diagnosis	Morphology	IHC (T-cell population of interest)	Flow (T-cell population of interest)	Gene rearrangements		Peripheral blood CBC with 100-cell differential count (%)^∗^	
Left iliac crest 0 days	T-cell ALL	Small- to medium-sized blast population comprising 95% of cells; marrow nearly 100% cellular	Positive: TdT, beta F1 Negative: TCR*γ*	Positive: CD3, CD4/CD8 dual positive, CD2, CD5 (d), CD7, and TDT (d) Negative: CD1a, CD34, CD117	T*β*A	257	WBC^a^	18.7
					T*β*B	269	Hemoglobin^b^	10.5
					T*β*C	182.9, 190.84	MCV^c^	92.8
					T*γ*A	190.94, 214.3	Platelets^a^	211
					T*γ*B	172.9	Lymphocytes	15
							Atypical lymphocytes	0
							Blasts	1
Left iliac crest 104 days	No evidence of malignancy	nc	np	nc	T*β*A	256	WBC^a^	3.62
					T*β*B	126, 263.8	Hemoglobin^b^	10.8
					T*β*C	190.63	MCV^c^	92.8
					T*γ*A	Germline	Platelets^a^	334
					T*γ*B	172.4	Lymphocytes	37
							Atypical lymphocytes	0
							Blasts	0
Cerebrospinal fluid 120 days	T-cell ALL	Large granular lymphocyte-like cells with condensed chromatin, inconspicuous nucleoli, eccentric nuclei, and moderate pale blue cytoplasm with prominent azurophilic granules	np	Positive: CD3c, CD7, CD8, CD45 (b), CD4 equivocal Negative: CD3, TdT, CD34	np		WBC^a^	2.44
							Hemoglobin^b^	9.5
							MCV^c^	88.3
							Platelets^a^	104
							Lymphocytes	34.4
							Atypical lymphocytes	0
							Blasts	0
Right iliac crest 124 days	*γδ* PTCL	Atypical lymphohistiocytic T-cell infiltrate with granulomas; large mononuclear lymphocytes with round to irregular nuclear contours and moderate eccentric light blue pale cytoplasm and cytoplasmic granules	Positive: CD2, CD3 (>50%), CD5, CD7 (f), CD8, CD56, perforin, TIA, CD4 (equivocal) Negative: TdT, CD117, CD68, CD34, CD1a, CD99, granzyme-B, CD30, CD57, CD25, ISH EBER	Positive: CD3, CD3c, CD5, CD7, CD4 (d), CD8 (d), CD45 Negative: TdT, CD34, CD56	T*β*A	275	WBC^a^	2.56
					T*β*B	126, 258, 263.8	Hemoglobin^b^	8.7
					T*β*C	182.92, 190.63	MCV^c^	88.1
					T*γ*A	214.13	Platelets^a^	76
					T*γ*B	172.48	Lymphocytes	28.9
							Atypical lymphocytes	0
							Blasts	0
Skin, right arm 125 days	*γδ* PTCL	Atypical lymphoid cells	Positive: CD2, CD3, CD5, CD4, CD8, CD56, TIA, CD99 (d), granzyme-B, TCRγ Negative: CD7, TdT, CD34, CD1a	nd	np		WBC^a^	2.61
							Hemoglobin^b^	9.8
							MCV^c^	87.9
							Platelets^a^	98
							Lymphocytes	32.2
							Atypical lymphocytes	0
							Blasts	0
Skin, right flank 189 days	γδ PTCL	Atypical lymphocytic infiltrate comprised of large pleomorphic with round to irregular nuclear contours and ample cytoplasm with prominent nucleoli and elevated N:C ratio	Positive: CD3, CD4, CD8, TCRγ, CD56 Negative: TdT, Beta F1, CD20	Positive: CD3, CD5, CD4/CD8 dual positive, CD45, CD56 Negative: CD7 (partial loss)	np		WBC^a^	9.1
							Hemoglobin^b^	10.9
							MCV^c^	95.8
							Platelets^a^	58
							Lymphocytes	8
							Atypical lymphocytes	0
							Blast	0

Note: (b) bright; (d) dim; (f) focal; (s) positive in subset. +/− equivocal; nc, noncontributory; nd, nondiagnostic due to insufficient specimen; np, not performed. ^a^In k/*μ*L; ^b^in g/dL; ^c^in fL. ^∗^Manual differential used when available; otherwise, automated impedance counts were utilized.

**Table 2:** Patient 2 pathological findings, by site.

	Site (days from original diagnosis)	Diagnosis	Morphology	IHC (T-cell population of interest)	Flow (T-cell population of interest)	Gene rearrangements		Peripheral blood CBC with 100-cell differential count (%)^∗^		Cytogenetics
5/2009	Mediastinal mass	T-cell ALL	Monotonous population of immature lymphoid cells with high N:C ratio, round to oval nuclei, mild nuclear irregularity, and scant cytoplasm	Positive: TdT (90%)	Positive: CD2, CD3, CD5 (d), CD7, CD4, CD8, CD45, CD10 Negative: CD20, EpCAM, Cytokeratin	T*β*A	Germline	WBC^a^	6.4	
						T*β*B	Germline	Hemoglobin^b^	12.8	
						T*β*C	Germline	MCV^c^	87.2	
						T*γ*A	Germline	Platelets^a^	209	
						T*γ*B	185.23, 193.82	Lymphocytes	18.7	
								Atypical lymphocytes	0	
								Blasts	0	
	Pleural fluid 17 days	T-cell ALL	Immature lymphoid blasts	Positive: CD3, CD4, CD8, TdT, CD99 Negative: CD79a	np	np		nc		No mitotic activity
	Right iliac crest 18 days	T-cell ALL	Sheets of lymphoblasts with high N:C ratio, immature chromatin, visible nucleoli, scant cytoplasm with occasional cytoplasmic vacuoles	np	Positive: CD45 (d), cyCD3, CD7, CD4, CD8, TdT (d), CD117, CD10 Negative: CD20, CD34; loss of surface CD3 and CD5	np		WBC^a^	9.94	
								Hemoglobin^b^	13.9	
								MCV^c^	86.2	
								Platelets^a^	262	
								Lymphocytes	Few	
								Atypical lymphocytes	22	
								Blasts	0	
9/2009	Left iliac crest 131 days	Normocellular marrow with NEM	nc	nc	nc	Germline		nc		Normal
3/2013	Right iliac crest^†^ 1417 days	Normocellular marrow with NEM	nc	np	nc	T*β*A	262.3	WBC^a^	6.39	
						T*β*B	261.10, 266.54	Hemoglobin^b^	1.7	
						T*β*C	188.74	MCV^c^	90.6	
						T*γ*A	Germline	Platelets^a^	165	
						T*γ*B	174.32	Lymphocytes	19.7	
								Atypical lymphocytes	0	
								Blasts	0	
	Pleural fluid 1419 days	PTCL, NOS with 75% large T-cells	Abundant large lymphoid cells with condensed chromatin, markedly irregular nuclear contours, frequent horseshoe-shaped nuclei, occasional binucleation, inconspicuous nucleoli, moderate-to-abundant pale blue cytoplasm, and cytoplasmic azurophilic granules	np	Positive: CD3c, CD8, CD45 Negative: CD3, CD7, CD4, TdT, CD117, CD20, CD34, CD56	T*β*A	259.6 (+/−)	WBC^a^	6.12	SNP ARRAY
						T*β*B	252.79, 272.24	Hemoglobin^b^	10.4	
						T*β*C	326.3	MCV^c^	91.5	
						T*γ*A	208.3, 216.77	Platelets^a^	190	
						T*γ*B	Germline	Lymphocytes	19.6	
								Atypical lymphocytes	0	
								Blasts	0	
3/2013	Left chest wall mass 1419 days	PTCL, NOS^∗∗^	Skeletal muscle extensively involved by a diffuse infiltrate of large lymphoid cells with granular chromatin, marked nuclear pleomorphism, markedly irregular nuclear contours, occasional horseshoe-shaped nuclei, and occasional conspicuous nucleoli	Positive: CD2, CD3, CD8 (b), ki67 (80%) Negative: CD5, CD7, CD4, TdT, CD15, CD20, CD30, CD34, CD56, pan-keratin, PAX5, EBV-LMP, EBER	np	np		nc		
	Left flank mass	PTCL, NOS	Large lymphoid cells with granular chromatin, marked nuclear pleomorphism, markedly irregular nuclear contours, occasional horseshoe-shaped nuclei, occasional conspicuous nucleoli, moderate pale eosinophilic cytoplasm, and abundant cytotoxic granules	Positive: CD3 (b), granzyme-B Negative: CD5, TdT, CD20, CD30, CD34, ALK-1, CD1a, myeloperoxidase, EBER ISH	Not representative					

Note: (b) bright; (d) dim; (f) focal; (s) positive in subset; +/− equivocal. NEM, no evidence of malignancy; nc, noncontributory; nd, nondiagnostic due to insufficient specimen; np, not performed. ^a^In k/*μ*L; ^b^in g/dL; ^c^in fL. ^∗^Manual differential used when available; otherwise, automated impedance counts were utilized. ^†^The IHC profile is from a chest wall mass biopsy taken on the same day. This biopsy had a dense atypical lymphocytic infiltrate. ^∗∗^Flow photocytometry demonstrated a 5% population of small T-cells. Limited material hindered full evaluation.
